# Supplementary material for: Impact of agricultural management on bacterial laccase-encoding genes with possible implications for soil carbon storage in semi-arid Mediterranean olive farming
Source: PeerJ. 2016 Jul 21;4:e2257. doi: 10.7717/peerj.2257 (PMC4963216; doi:10.7717/peerj.2257)
Supplement: Supplemental Information 1 [file peerj-04-2257-s001.docx]

The study was carried out at the Finca Salido Bajo in Jaen, in southeast Spain (38º 12 36 N 03º 23 01W).

Average annual rainfall for the area is 557 mm. The average maximum temperature is 37.1 ºC and the average minimum is 2.8 ºC. Parent material is a red clastic belonging to the Buntsandstein facies and made up basically of alluvial sandstone, clay and silt lenses together with some conglomerates. The common soil throughout the plantation is Anthropic Regosol (FAO, 1998). It is characterised by the presence of alternate layers of sandstone and clay that had been greatly modified by tillage prior to the experiment. At a depth of 40–50 cm (depending upon the intensity of erosive processes), there are laminar layers of highly consolidated sandstone. These layers restrict water infiltration, resulting in periods of prolonged surface flooding after rain. Sometimes, these periods can last for up to several weeks depending upon the intensity of the rainfall and/or evaporative demands.
