# Supplementary figures and images for: Impact of agricultural management on bacterial laccase-encoding genes with possible implications for soil carbon storage in semi-arid Mediterranean olive farming
Source: PeerJ. 2016 Jul 21;4:e2257. doi: 10.7717/peerj.2257 (PMC4963216; doi:10.7717/peerj.2257)

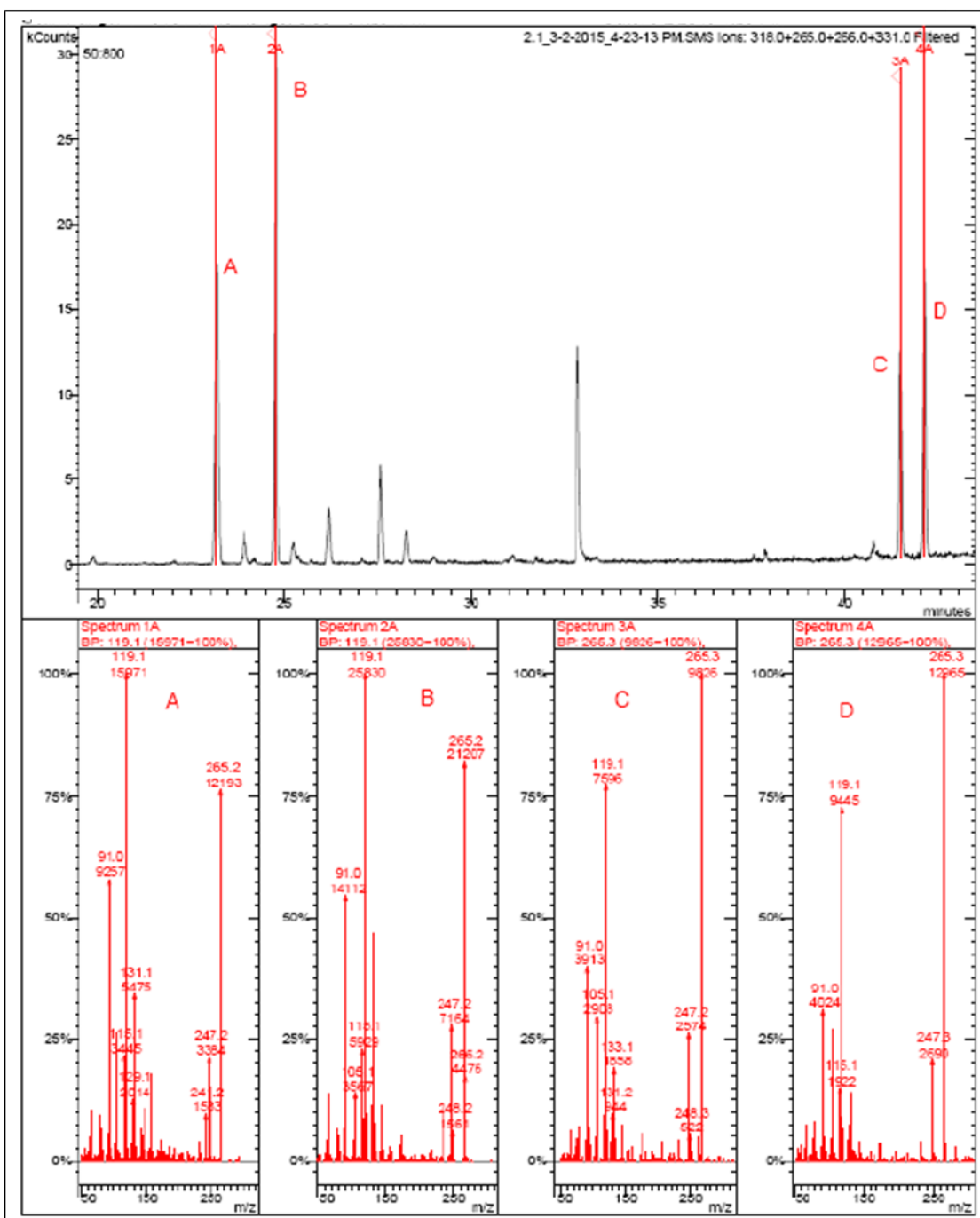

Supplement: Supplemental Information 2 [file peerj-04-2257-s002.pdf]

DNA-based DGGE:

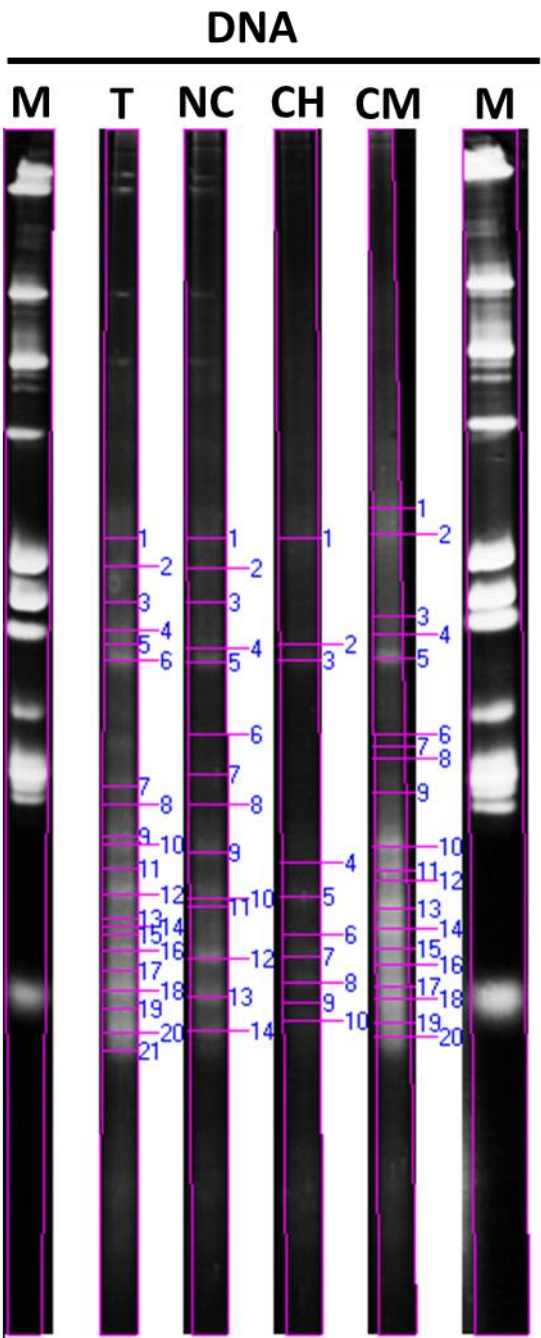

RNA based DGGE:

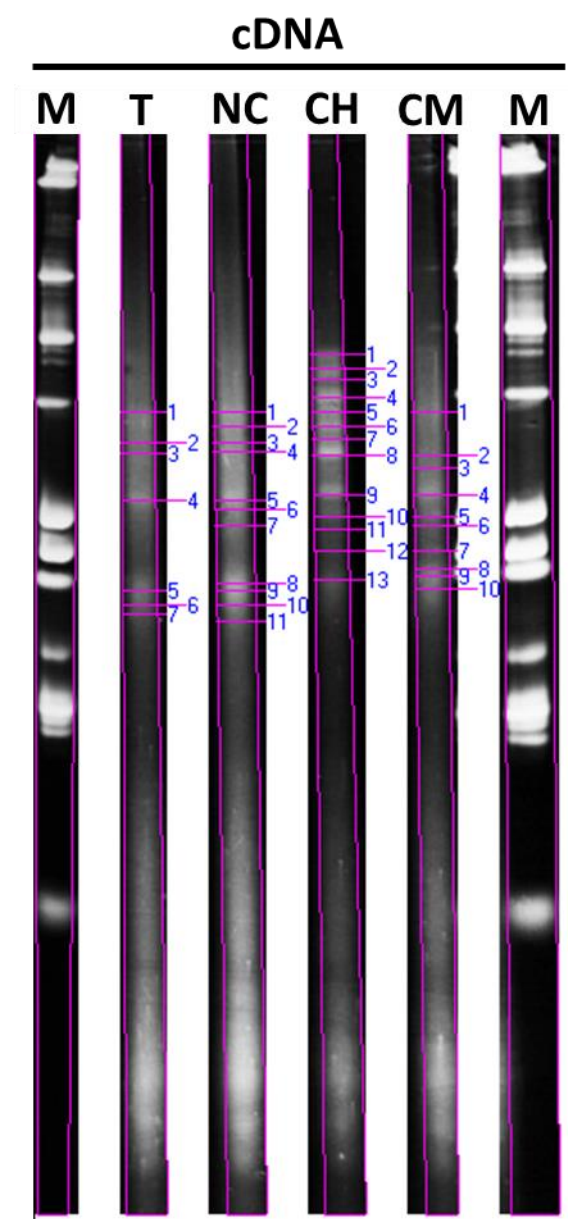

Supplement: Supplemental Information 4 — Computer-generated DGGE image used by the UVItec Gel Documentation software for analysing the bands. [file peerj-04-2257-s004.pdf]
