# Supplementary material for: Impact of agricultural management on bacterial laccase-encoding genes with possible implications for soil carbon storage in semi-arid Mediterranean olive farming
Source: PeerJ. 2016 Jul 21;4:e2257. doi: 10.7717/peerj.2257 (PMC4963216; doi:10.7717/peerj.2257)
Supplement: Supplemental Information 3 — Checking the homology of Cu1AF-Cu2R primers to anneal and amplify the target DNA. [file peerj-04-2257-s003.docx]

| **Homology found with the primers Cu1AF-Cu2R to amplify LMCO-genes** | | |
| --- | --- | --- |
| **AMINO ACID SEQUENCE** | **Nº of clones** | **Highest match in the database (% identity amino acids)** |
| TTVHWHGVRLPNAMDGVPGLTQPPIKPGEQFTYEFTPLDAGTFWYH | 1 | ref\|WP_007602744.1\| Bradyrhizobium sp. WSM1253. Copper oxidase (96%) |
| TTVHWHGVRTPSPMDGVPGLSFPGIAPGETFIYRFPVHQSGTFWYHS | 3 | gb\|ABQ52278.1\| Uncultured bacterium. Laccase (95%) |
| TSVHWHGIILPNPMDGVPGLTFHGIAPGETFTYQIPVRQSGTFWYHS | 2 | gb\|ABQ52292.1\| Uncultured bacterium. Laccase (95%) |
| TSVHWHGIRLPNAMDGVPDLTQKPISSGERFDYAFTPPDAGTFWYHS | 3 | gb\|ABQ52259.1\| Uncultured bacterium. Laccase (95%) |
| TVHWHGILLPANMDGVPGLSFHGIQPGDTYVYRFQVRQAGTFWYHS | 1 | gb\|ADH15950.1\| Uncultured bacterium. Putative laccase (93%) |
| TSVHWHGMILPANMDGVPGISFPGIRPGETHEYRFPVTQAGTFWYHS | 3 | gb\|ABQ52303.1\| Uncultured bacterium. Laccase (94%) |
| TVHWHGIRAPSDMDGVPGLSFPGIAPGETFVYRFPVRQSGTFWYHS | 1 | gb\|ADH16167.1\| Uncultured bacterium. Putative laccase (93%) |
| TTVHWHGIILPNPMDGVPGLTFQGIAPGETFTYQFPVRQSGTFWYHS | 1 | gb\|ABQ52332.1\| Uncultured bacterium. Laccase (87%) |
| TSVHWHGVLVPFAMDGVPGVSFPGISPGETFAYEFPVIQSGTFWYHS | 1 | ref\|YP_002130318.1\| Phenylobacterium zucineum HLK1. Copper-binding protein (87%) |
| TSVHWHGILVPAGMDGVPGLSFDGIAPGETFVYRFEVKQSGTFWYHS | 1 | emb\|CAJ77138.1\| Agromyces salentinus. Laccase-like multicopper oxidase (87%) |
| TSVHWHGVIVPADMDGVPGLSFSGIGPGETFVYRFRLNQSGTFWYHS | 1 | gb\|ABQ52289.1\| Uncultured bacterium. Laccase (87%) |
| TVHWHGLRIPSAMDGTEVVQRAIQPGETFTYRFTPPDAGTFWYHS | 1 | gb\| ADH16044.1\| Uncultured bacterium. Putative laccase (87%) |
| TTVHWHGLLVPFAMDGVPGISFPGINPGETFVYEFPVIQSGTFWYHS | 2 | ref\|YP_007617272.1\| Sphingomonas sp. MM-1. Putative multicopper oxidase (87%) |
| TSVHWHGVRSPAGMDGVPGLSFPGIAPGETFTYRIPIHQSGTFWYHS | 1 | gb\|ABQ52287.1\| Uncultured bacterium. Laccase (87%) |
| TSVHWHGVRVPNGMDGVNGLTQPTIDPGQTFRYEFTVPDAGTFWYHS | 1 | gb\|ABQ52315.1\| Uncultured bacterium. Laccase (85%) |
| TTVHWHGLHLPPEQDGASEEGSPIIAPGGSLVYAFTPKPSGTFWYHS | 1 | gb\|ABQ52246.1\| Uncultured bacterium. Laccase (83%) |
| TSVHWHGIRVPNGMDGANGITQPPIEPGQTFRYEFTVRD | 1 | gb\|ABQ52311.1\| Uncultured bacterium. Laccase (82%) |
| TTVHWHGLLVPNPMDGVPGVNFGGIRPGETFTYRFPLQQYGTFWYHS | 1 | gb\|ABQ52339.1\| Uncultured bacterium. Laccase (81%) |
| TSVHWHGLILPSSQDGVPGVSDGFQGIPPGQTFTYRFPVRQSGTFW | 1 | gb\|ABQ52299.1\| Uncultured bacterium. Laccase (80%) |
| SVHWHGIELESYYDGVPHWNGDDRRRTPSIEPNQQFVARFTPPRAGTFWYHS | 1 | gb\|ADH15981.1\| Uncultured bacterium. Putative laccase (75%) |
| SVHWHGIRVPDAEDGVAGLTQNAVAPGESFTYEFVARDAGTFWYHS | 1 | gb\|ADH16126.1\| Uncultured bacterium. Putative laccase (74%) |
| TTVHWHGIPIINTMDGVPGITQPAIKAGTNFLYDFTVPVSG | 2 | ref\|YP_002823063.1\| Sinorhizobium fredii NGR234. Multicopper oxidase (74%) |
| TSVHWHGIELDNQADGTPYTQNQVPQNGTFLYKFKVDRPGTFWYHS | 1 | gb\|ABQ52345.1\| Uncultured bacterium. Laccase (72%) |
| TTVHWHGLAIRNDMDGAPEVTQPGIATGQSAVYEFTLPHPGTFWYH | 1 | ref\|WP_017882576.1\| Leucobacter sp. UCD-THU. Copper oxidase (70%) |
| TSVHWHGLMVPSIEDGVPGVGQKPIQAGQTYVYDFTVHDQDVGTFWYHS | 4 | gb\|ADV52165.1\| Uncultured bacterium. Laccase (63%) |
| SVHWHGLDMPSALDG-DPMTAPGGHSVLQGKTFLYHFVAKYPGTFWYHS | 1 | gb\|ADH16113.1\| Uncultured bacterium. Laccase (55%) |
